# Polymeric Materials Selection for Flexible Pulsating Heat Pipe Manufacturing Using a Comparative Hybrid MCDM Approach

**DOI:** 10.3390/polym15132933

**Published:** 2023-07-03

**Authors:** Muhammed Ordu, Oguzhan Der

**Affiliations:** 1 Department of Industrial Engineering, Faculty of Engineering, Osmaniye Korkut Ata University, 80010 Osmaniye, Turkey; 2 Department of Marine Vehicles Management Engineering, Faculty of Maritime, Bandirma Onyedi Eylul University, 10200 Balikesir, Turkey; oder@bandirma.edu.tr

**Keywords:** flexible pulsating heat pipe, polymer, material selection, multi-criteria decision-making

## Abstract

The right choice of polymeric materials plays a vital role in the successful design and manufacture of flexible fluidic systems, as well as heat transfer devices such as pulsating heat pipes. The decision to choose an acceptable polymeric material entails a variety of evaluation criteria because there are numerous competing materials available today, each with its own properties, applications, benefits, and drawbacks. In this study, a comparative hybrid multi-criteria decision-making (MCDM) model is proposed for evaluating suitable polymeric materials for the fabrication of flexible pulsating heat pipes. The decision model consists of fourteen evaluation criteria and twelve alternative materials. For this purpose, three different hybrid MCDM methods were applied to solve the material selection problems (i.e., AHP-GRA, AHP-CoCoSo, and AHP-VIKOR). According to the results obtained, PTFE, PE, and PP showed promising properties. In addition, Spearman’s rank correlation analysis was performed, and the hybrid methods used produced consistent rankings with each other. By applying MCDM methods, it was concluded that PTFE is the most suitable material to be preferred for manufacturing flexible pulsating heat pipes. In addition to this result, PE and PP are among the best alternatives that can be recommended after PTFE. The study supports the use of MCDM techniques to rank material choices and enhance the selection procedure. The research will greatly assist industrial managers and academics involved in the selection process of polymeric materials.

## 1. Introduction

Electronic devices are undergoing significant shape and performance changes as a result of technological advancement [1]. Especially in recent years, the demand for flexible electronic devices, such as flexible display panels and wearable devices, has been increasing rapidly. One of the main reasons for the problems encountered by flexible electronic devices with these performance characteristics is the problem of overheating. In terms of being both a solution to this problem and fulfilling the desired flexibility criteria, there is a serious need for an advanced heat spreader that is flexible, thin, and has high thermal performance. The pulsating heat pipe (PHP), which is formed by curling a smooth tube, stands as an encouraging solution for the future because of its simple and wick-free design [2,3]. A thermally conducive fluid, devoid of non-condensable gases and possessing optimum thermophysical characteristics, is then introduced into the system, thereby only partially populating the device. Once the integrity of the PHP is assured through sealing, an intriguing dynamic commences, with the heat transfer fluid transitioning between liquid slugs and vapor plugs. This behavior is facilitated by the preponderance of capillary forces over gravitational forces. Although the fluid’s trajectory and the resulting flow patterns appear to be largely stochastic, the system remarkably attains a quasi-steady state across an extensive range of operating parameters [4]. Internal pressure perturbations within the PHP trigger self-induced oscillatory kinetics involving liquid plugs and vapor bubbles [5], which considerably accentuates convective heat transfer. Consequently, this optimizes the heat exchange process, encompassing both latent and sensible heat between a thermal source (termed as the evaporator) and a thermal drain (the condenser) [6]. When juxtaposed with traditional heat transfer modalities, such as purely conductive systems or single-phase forced circulations, PHPs exhibit distinct advantages. These primarily stem from the exploitation of heightened heat transfer rates associated with phase transition phenomena, compactness, and the capability for passive operation. The latter obviates the need for mechanical pumps or gravity dependence, thus further underscoring the utility and practicality of PHPs [7].

In the last forty years, much research has been carried out to understand the physical and operational properties of PHPs. In almost all of these studies, PHPs were produced using hard materials such as copper, aluminum, glass, and silicon. The main reason for using these materials is that they have good gas barrier properties. However, they are not suitable materials for the production of these heat spreaders, which are planned to be used in flexible devices [8].

Polymers are suitable materials for the production of flexible PHPs because of their high flexibility. Some researchers realized the importance of this situation and produced PHPs using polymeric materials. The first polymeric PHP was produced with channels that were 50 mm wide, 56 mm long, and had a diameter of 2 mm. In this study, polydimethylsiloxane (PDMS) was chosen as the basic production material, and two types of working fluids (methanol and ethanol) were used [9]. PDMS-based PHPs of a similar size were produced and the effect of nanofluid under an electric field on the thermal performance of PHPs was studied in detail [10]. In addition, thin and transparent PHPs, using polyethylene terephthalate polymer for the PHP casing and UV-curable polymer resins for the PHP channel, were improved by [11]. Although these studies focused on flexible PHPs, metal pipes or plates were used in the evaporator and condenser parts, and the flexibility was partially lost.

Recently, a low-density polyethylene sheet was cut into pieces and an aluminum layer was placed on both sides of the sheet to act as a gas barrier. With this layer, a flexible PHP production was manufactured by sintering the aluminum-polymer film. An indium coating was placed around the heat pipe to minimize the diffusion of non-condensable gases [12]. In another recent study, flexible PHP was produced using CO_2_ laser cutting and fiber laser welding. In production, only polypropylene materials of different thicknesses and colors were used, and a 100% polymeric pulsating heat pipe was produced without using any metallic materials [13].

From the selection of the most suitable material to the determination of the most suitable manufacturing process parameters, there are many decision-making points that can affect the output factors. However, the fact that cost- and benefit-oriented criteria have to be taken into account in the same problem complicates the decision-making process in determining the most suitable material or parameter. To overcome this, multi-criteria decision-making techniques have been successfully applied. A number of studies have focused on optimizing the machining parameters. For example, a novel decision support tool was developed to optimize the machining parameters of AISI 4140 steel by using fuzzy MCDM methods [14]. CNC turning parameters were optimized using six MCDM methods (i.e., WASPAS, EDAS, TOPSIS, MOORA, VIKOR, and MABAC) in a fuzzy environment [15]. Furthermore, MCDM techniques were successfully applied to optimize the drilling process of multiwall carbon nanotube/epoxy nanocomposites by [16], machinability of Nimonic C-263 superalloy by [17], machining AISI O1 tool steel by [18], a micro-electrical discharge machining process by [19], drilling parameters of glass fiber reinforced polymer by [20], cutting parameters in milling processes by [21], and milling parameters of aluminum 1100 alloy by [22]. On the other hand, reliable and accurate decisions were aimed for in material selection problems by using MCDM methods. Another issue with the usage of MCDM techniques in the manufacturing process is determining suitable materials according to the intended use. For instance, the techniques were applied with the aim of determining the most energy-efficient material, considering the environmental effects [23], specifying the brake booster valve body [24], choosing materials suitable for additive manufacturing technologies [25], and selecting 3D-printed polyamide-based composites for automobile front bumpers [26].

Composites and polymeric materials have also occasionally caused issues with material choices. For example, an experimental process was conducted; then, MCDM techniques were applied for calculating criteria weights, and the most suitable polymeric nanocomposite material to be used in automotive bumper beams was determined [27]. Data envelopment analysis (DEA), AHP, and TOPSIS methods were integrated to determine the most suitable polymeric material for cutting, based on the output parameters obtained from the CO_2_ laser-cutting process of three different polymeric materials (i.e., polyethylene, polycarbonate, and polypropylene). It was mentioned that polymeric materials for laser beam machining might be selected, depending on material type and thickness [28]. Only the preference selection index (PSI) was applied to specify the best polymer matrix composite (PMC) that was resistant to impact loading. The twelve polymer alternatives were assessed under five criteria (i.e., density, tensile strength, tensile modulus, cost, and flexibility), and the fiber alternatives using the above criteria (including cellulose instead of flexibility as a criterion) were also compared [29].

The literature parts described above proved the value of MCDM techniques in the choice of material procedure. However, it seems that there is not yet a study focusing on the selection of the most suitable polymeric material for various flexible heat transfer and fluid devices. However, using a variety of hybrid MCDM techniques, the most suitable material for various flexible heat transfer and fluidic devices can be assessed and chosen, one of which is the use of a hybrid MCDM to determine the best material for flexible PHPs. For the fabrication of flexible PHPs, the following polymeric materials were used as alternatives, by taking into account the literature: Acrylonitrile butadiene styrene, Polyamides (Nylons), Polycarbonate, Polyetheretherketone, Polyethylene, Polyethylene terephthalate, Polymethyl methacrylate, Polyoxymethylene (Acetal), Polypropylene, Polystyrene, Polyvinylchloride, and Polytetrafluoroethylene (Teflon). Unfortunately, there have not been many products created entirely from polymeric materials. The decision-making process increases the complexity of suitable material selection by taking the evidence into consideration. The development and implementation of hybrid MCDM approaches for choosing the best thermoplastic material for flexible PHPs was the main focus of this paper. To do this, we applied a three-stage approach. Then, after identifying the options and criteria, the criteria weights were determined using the AHP approach. After that, three MCDM methods (i.e., GRA, CoCoSo, and VIKOR) were employed to rank the alternatives from the most suitable to the least suitable by integrating the criteria weights. In the last stage, Spearman’s rank correlation coefficients were calculated to better understand the consistency of MCDM methods with each other, in terms of the rankings they produced. Manufacturing PHPs is very laborious and costly, and it is necessary to spend a lot of time selecting the material to be used. Thus, this study was carried out to eliminate this wasted time and to simplify the material selection process for flexible PHPs or different types of flexible fluidic and heat transfer devices.

The remains of the paper are organized as follows: Section 2 explains the selected alternatives and criteria and describes the comparative hybrid MCDM approach used in the study and the methodological aspects of each MCDM method. Section 3 discusses the results, and the study is concluded in Section 4.

## 2. Materials and Methods

### 2.1. Material Alternatives

Below are some brief key features of the 12 different types of thermoplastic materials that can be used in the manufacture of flexible PHPs and other different types of flexible heat transfer and fluidic devices. The twelve alternative thermoplastics and their abbreviations are provided in Table 1. Acrylonitrile butadiene styrene (ABS) is a type of polymer that can be found in both light and hard forms and is widely used in products produced by molding. Moreover, it offers excellent thermal stability, stiffness, chemical resistance, and crack resistance due to environmental stress. ABS is affordable, lightweight, flexible, and simple to extrude. Communication devices, automotive interiors, luggage, toys, and boats are some of the common areas in which ABS is used [30]. Nylon is a type of polyamide thermoplastic. It was developed in the mid-1930s and has since been used in almost every industry. Some of the areas in which nylon is most used are: gears, bearings, sanitary ware, packaging, bottles, fabrics, textiles, and rope manufacturing. The group of thermoplastic polymers known as polycarbonates (PC) includes compounds with carbonate groups in their chemical structure. Polycarbonates generally used in manufacturing are strong, durable, and optically transparent. This transparent material has exceptional physical qualities, including high impact resistance, extremely good heat resistance, excellent toughness, good electrical qualities, good dimension stability, and high stiffness. Across a large temperature range, the characteristics are stable. Other notable features are that they are easily machined, molded, and thermoformed. Due to these characteristics, polycarbonates find applications in many different areas, such as in safety glasses, shields, helmets, lighting fixtures, and medical components [31]. A colorless organic thermoplastic polymer from the polyaryletherketone family, polyether ether ketone (PEEK), is used in engineering applications. Because of its exceptional mechanical, chemical, and thermal qualities, it is employed in a wide range of applications, particularly in the aerospace, automotive, and electronics industries. PEEK is also frequently utilized in the medical sector for dental materials and bone restoration, due to its high biocompatibility and high thermal resistance [32]. Thermoplastic polyethylene (PE), which has a flexible crystal structure, is a lightweight, strong material. PE is one of the most produced plastics in the world. They can be used in various applications, such as packaging, bags, squeezing tubes, toys, and artificial joints [33]. Polyethylene terephthalate (PET) is a thermoplastic polymer resin from the polyester family of resins. Some of the most common areas of use are blow-molded bottles, film, audio/video cassettes, sails, and sail accessories. Polymethyl methacrylate (PMMA) is a synthetic resin produced from the polymerization of methyl methacrylate. PMMA is strong and stiff, with excellent weather resistance. The substance is as transparent as glass and is frequently utilized in optical applications. It has strong flexural and tensile characteristics. Ten-times more impact resistant than glass, PMMA is the most common thermoplastic, with the highest surface hardness and scratch resistance. PMMA is mostly used as a glass replacement in products such as unbreakable windows, skylights, illuminated signs, and aircraft canopies [31]. Acetal, also known as polyoxymethylene (POM), is a thermoplastic that is used to make precision parts with high stiffness, low friction, and excellent dimensional stability. Zippers, household and appliance parts, and handles are some of the most basic uses. A thermoplastic polymer called polypropylene (PP) is used in a wide range of applications. It is produced from the monomer propylene through chain-growth polymerization. It is one of the lightest thermoplastics on the market and can be utilized as a fiber and as a structural plastic. It possesses great rigidity, abrasion resistance, good strength, even at quite high temperatures, good elastic qualities, and a hard, glossy surface. Without deforming, PP can withstand temperatures of roughly 160 °C. The most common usage areas are, respectively, ropes, garden furniture, pipes, water heaters, electrical insulation, and astroturf turf [31]. The synthetic aromatic hydrocarbon polymer known as Polystyrene (PS) is created from the styrene monomer. Polystyrene can be solid or foamed. Generally speaking, polystyrene is clear, hard, and brittle. Per unit weight, it is a reasonably priced resin. It has a relatively low melting point and a poor barrier to oxygen and water vapor. PS is widely known for its resistance to alkaline, diluted/concentrated acids, alcohols, and polar solvents. Polystyrene is one of the most widely used plastics and its production scale is several million tons per year. Polystyrene can be naturally transparent or colored with colorants. Toys, packaging, cutlery, and audio cassette/CD boxes are some of the most common uses [34]. Polyvinyl chloride (PVC) is the world’s third most widely produced synthetic plastic polymer (after polyethylene and polypropylene). PVC film has the benefits of being inexpensive, robust, transparent, opaque, very insulating, waterproof, and anti-polluting. As a result, it is frequently used in electronics, food packaging, and medicine. Further, areas such as pipes, gutters, window frames, and packaging are where PVC is most commonly used [35]. Polytetrafluoroethylene (PTFE) is a synthetic tetrafluoroethylene fluoropolymer with numerous applications. The most commonly known name is Teflon. PTFE exhibits great resistance to corrosion, chemical reactions, high temperatures, and stress cracking. The most used application areas are non-stick coatings, mattresses, skis, electrical insulation, and tapes [36,37].

### 2.2. Criteria

It is a well-known fact that the production of flexible heat transfers or fluidic devices (e.g., flexible PHPs [13], serpentine channels [38]) is highly dependent on material properties. Therefore, the right choice of thermoplastic material is of great importance for the production of flexible devices at the desired level. Polymers are large molecules, or macromolecules, made up of many repeating subunits [39]. As a result of their molecular structure, polymer materials display specific physical features, such as toughness, viscoelasticity, and a tendency to form glass or semi-crystalline structures [40]. The most distinctive features that make polymeric materials more advantageous than silicon, glass, metal, and aluminum are their low cost, excellent corrosion resistance, durability, and recyclability, as well as their suitability for producing flexible fluidic and heat transfer devices [41]. To select the thermoplastic materials that should be chosen for the manufacture of flexible heat transfer and fluidic devices, a clear understanding of the application requirements must first be made.

The density of the polymer often depends on its crystallinity. The density of the material is important in determining whether it is an amorphous or crystalline polymer. Although it does not directly affect the material selection, it is important to know the densities of the polymers to be selected. Another important criterion in material selection is the yield strength of the plastic. Before the material yields strength, it will behave elastically. In other words, if the strain applied to the material is stopped at the elastic part, the material can return to its original shape. When it reaches the yield strength of the plastic, it does not return to its former size and begins to stretch. For the production of a flexible and durable PHP, the yield value of the selected material is expected to be as high as possible. If this situation cannot be achieved, it may cause a change in the dimensions of the material used in its manufacture due to plastic deformation. The other significant criterion is the maximum stress that a material can withstand while being stretched or pulled before breaking, which is called its tensile strength. Generally, the ultimate tensile strength in brittle materials is close to the yield point. On the other hand, the ultimate tensile strength may be higher in ductile materials. As with the yield value, it is desirable that the tensile strength value be as high as possible in the flexible PHP design. The flexibility of the material is another crucial selection factor. To determine whether the elasticity is suitable, its Young’s modulus and flexural modulus should be as low as is feasible [42].

Whether or not a thermoplastic has adequate ductility for the application is a key consideration when choosing the best thermoplastic. The capacity of a material to deform without breaking is known as ductility. One of the most basic methods to determine the ductility ratio is to establish the percent elongation of the tensile test specimen. In addition, ductile materials do not extend properly when loaded beyond necking. This is why it has become customary to express ductility in terms of % elongation relative to the length of an original material. The elongation, which is used to assess the material’s brittleness, is a crucial material selection factor. In general, polymeric materials with a small elongation are considered brittle. The elongation of the thermoplastic to be selected in the production of flexible PHP should be as large as possible. Fracture toughness is yet another crucial material characteristic. Briefly, it can be defined as the resistance to breakage when cracks are present in a material. In addition to being flexible, the fracture toughness of the material to be selected in the production of a durable PHP should be as high as possible. The bending modulus is a crucial mechanical characteristic. It is a dense property calculated as the stress-strain ratio in bending strain, or the tendency of a material to resist bending. The bending modulus of the material to be selected should be taken at the highest possible level for the production of suitable flexible devices [42].

The material’s specific heat capacity (Cp) should be taken into account as another crucial selection consideration. The specific heat capacity of a material is defined as the amount of energy required to raise its unit mass temperature by one degree. The specific heat capacity of the material to be selected should be as high as possible, in order to make the heat transfer efficient. Thermal conductivity (k) is an important value to be considered, showing the rate at which heat flows in or through a material. Compared to metallic materials, polymeric polymers typically have lower thermal conductivity [43,44]. The selection of materials with as high a thermal conductivity as possible is a desired feature for flexible heat and fluidic devices that are planned to be produced. Another critical parameter of thermoplastic material selection is thermal expansion, which refers to a property, such as shape, area, or volume, that changes when the temperature changes. The thermal expansion of the material can have some significant effects on the relative expansion or contraction of different materials, especially in assemblies such as electronics and computer components. The coefficient of thermal expansion is generally inversely proportional to the melting point of the material. The high thermal expansion of the thermoplastic to be selected for production is one of the main reasons for preference. Finally, the values to be considered are the maximum and minimum temperatures. The maximum temperature is important when materials are used at high temperatures for long periods of time, while the minimum temperature is vital so that the material does not become brittle. The operation of the material to be selected in the highest and lowest possible temperature ranges becomes important for the selection to be made [45,46]. The expected operating temperature range for the heat transfer equipment, specifically the flexible pulsating heat pipes (FPHPs), is considered to be −20 °C to 130 °C. This range has been chosen to ensure the safe and efficient operation of the PHP across a wide range of potential environments and applications. Furthermore, the broad range allows us to account for both the maximum operating limits of the materials and a margin of safety. The average physical, thermal, and mechanical properties for each of the thermoplastics are provided in Table 2.

The desired properties of the thermoplastic material to be selected for production were briefly mentioned above. In addition, the most important feature expected from the polymer to be selected is the affordable price. In addition, easy availability is another important feature that is desired. The selection criteria and abbreviations of the materials to be used in multi-criteria selection methods are shown in Table 3.

### 2.3. Comparative Hybrid MCDM Approach

**Figure 1:**
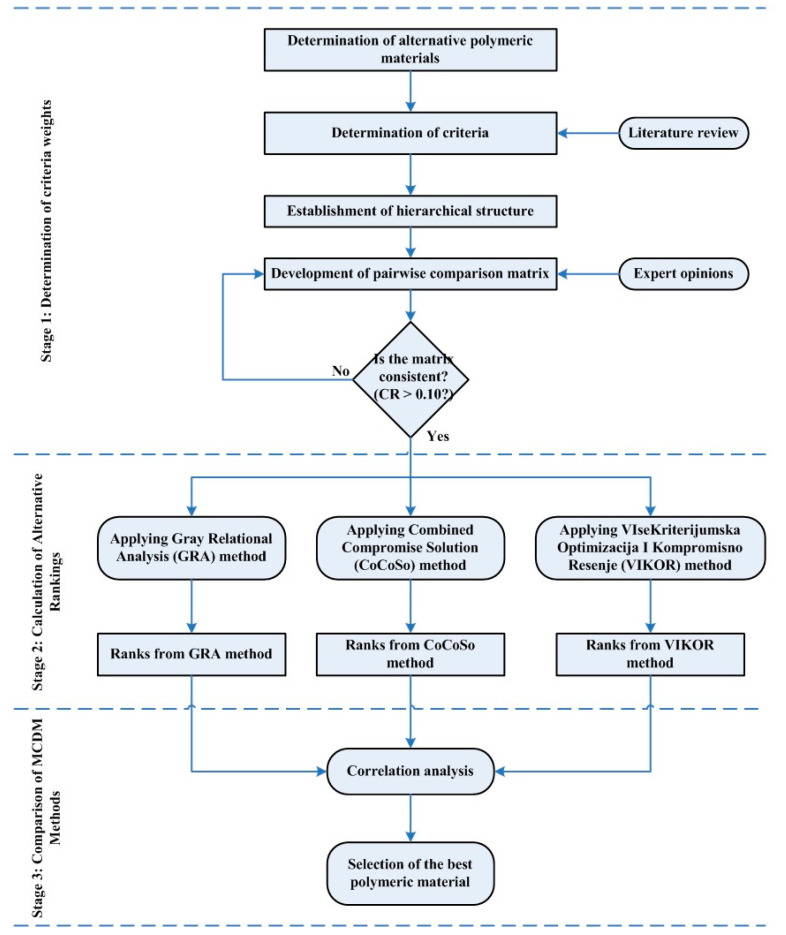
The comparative hybrid MCDM approach in the study.

**Figure 2:**
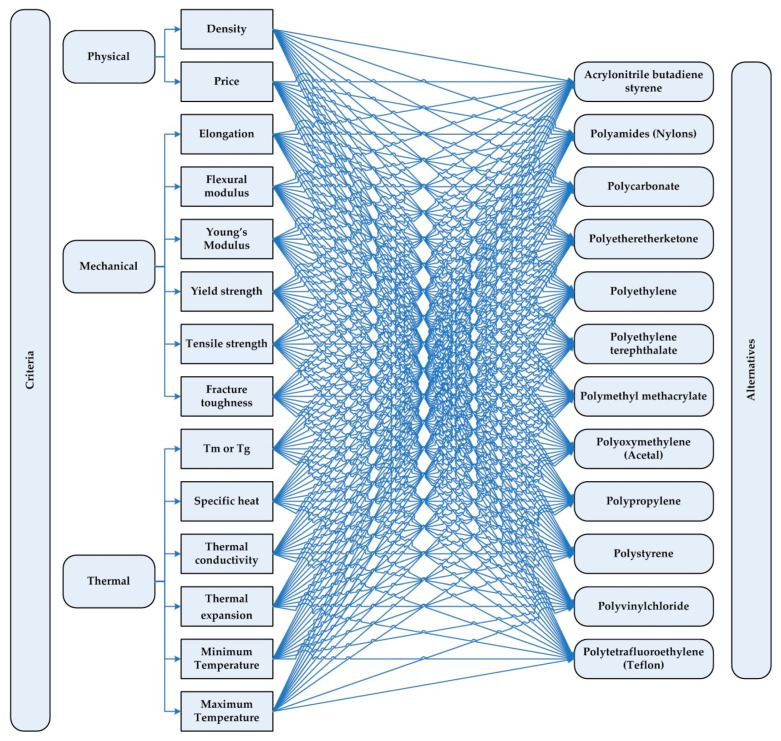
The hierarchical structure of the MCDM problem.

This paper has focused on the suitable polymeric material selection for manufacturing flexible PHP by applying a comparative hybrid MCDM approach, as seen in Figure 1. The hybrid approach consists of three stages: (1) determination of the criteria weights; (2) calculation of the alternative rankings; and (3) comparison of used MCDM approaches. In the first stage, the suitable polymeric materials to be used in flexible pulsating heat pipe manufacturing are determined as alternatives. After that, criteria used in the evaluation of alternatives are identified according to the literature. After establishing the hierarchical structure (see Figure 2), the pairwise comparison matrix is developed based on expert opinions. The second stage is carried out if the matrix is consistent. Otherwise, the pairwise comparison matrix is redeveloped by comparing the criteria with each other, and the consistency analysis is conducted again. In the second stage, the following MCDM approaches are performed by embedding the criteria weights from the AHP method: GRA, CoCoSo, and VIKOR. Thus, three different rankings are obtained. In the last stage, correlation analysis is carried out to better understand to what extent the rankings from the AHP-based MCDM approaches are consistent. In the end, the polymeric material that is most suitable for flexible PHP manufacturing is selected.

#### 2.3.1. Determination of the Criterion Weights by Analytical Hierarchy Proses (AHP) Method

One of the most popular methods to establish the criteria weight is the AHP method, which is used to tackle difficult problems requiring several criteria [48]. In this study, the following stages [49] of the AHP technique were used to solve the polymeric material selection problem.

The criteria and alternatives are what construct the hierarchical structure of the MCDM problem depicted in Figure 2. According to the experts’ evaluations of absolute numbers (see [50] for the definition and explanation of the absolute numbers), a pairwise comparison matrix is developed. Based on the pairwise comparison matrix, the relative normalized weights (w^1^) of each index and the weighted matrix are determined. The highest eigenvalue (€_max_), which is the average of the consistency values, is then derived after computing the consistency values. The consistency ratio is calculated at this point.

The consistency index (*CI*) is first calculated by using Equation (1):

(1) $$CI=\frac{\lambda_{max}-n}{n-1}$$

where *n* means the order of the pairwise comparison matrix, representing the number of criteria in the criteria layer, and $\lambda_{max}$ is the highest eigenvalue, which is the average of the consistency values.

The consistency ratio (*CR*) can also be calculated by using Equation (2):

(2) $$CR=\frac{CI}{RI}$$

where *RI* stands for the random index used in the literature [51]. If the consistency ratio (*CR*) is 0, that is, the consistency index is 0, the consistency of the pairwise comparison matrix is excellent [52] and the approved upper limit for *CR* is the value 0.1 [53]. This means that the pairwise comparison matrix becomes inconsistent as the consistency index increases. A new comparison matrix must be developed in order to increase consistency if the final consistency ratio is higher than this amount (i.e., 0.1) [53]. The random index provided calculates the average values of consistency index and depends on the order of comparison matrices [52].

#### 2.3.2. Gray Relational Analysis (GRA) Method

The Gray approach was developed by [54] to address uncertainty issues arising from incomplete and discontinuous data [55,56]. Moreover, the GRA technique is one of the most widely used approaches for examining numerous associations between discrete data sets and for making decisions when dealing with several attributes [57,58]. The main benefits of the GRA technique are that it is one of the greatest ways to make decisions in a corporate context, the calculations are easy to understand, and the conclusions are based on the original data [57,58]. The following is a summary of the processes [56]:

Make the data set normal. One of the three ways, namely larger-is-better, smaller-is-better, and nominal-is-best, can be used to treat data. In the transformation of larger-is-better, $x_{i}\left(j\right)$ can be transformed to $x_{i}^{*}\left(j\right)$. The definition of the formula is given in Equation (3):

(3) $$x_{i}^{*}\left(j\right)=\frac{x_{i}\left(j\right)-\underset{j}{\min}x_{i}\left(j\right)}{\underset{j}{\max}x_{i}\left(j\right)-\underset{j}{\min}x_{i}\left(j\right)}$$

where max $x_{i}\left(j\right)\;$ is the maximum value of entity *j* and $x_{i}\left(j\right)$^j^ is the minimum value of entity *j*. For smaller-is-better, use Equation (4) to transform $x_{i}\left(j\right)$ to $x_{i}^{*}\left(j\right)$:

(4) $$x_{i}^{*}\left(j\right)=\frac{\underset{j}{\max}x_{i}\left(j\right)-x_{i}\left(j\right)}{\underset{j}{\max}x_{i}\left(j\right)-\underset{j}{\min}x_{i}\left(j\right)}$$

For nominal-is-best, if the target value is $x_{0b}\left(j\right)$ and $\underset{j}{\max}x_{i}\left(j\right)\geq x_{0b}\left(j\right)\geq \underset{j}{\min}x_{i}\left(j\right)$, then the formula is as shown in Equation (5):

(5) $$x_{i}^{*}\left(j\right)=\frac{\left|x_{i}\left(j\right)-x_{0b}\left(j\right)\right|}{\underset{j}{\max}x_{i}\left(j\right)-x_{0b}\left(j\right)}$$

The referential series of *x*_0_ should also be standardized concurrently by either one of Equations (3)–(5). Here, $x_{0}\left(j\right)$ is utilized in place of $x_{i}\left(j\right)$. In the case of the transition, use larger-is-better. The normalized referential series of $x_{0}\left(j\right)$ is transformed to $x_{0}^{*}\left(j\right)$ by using Equation (6):

(6) $$x_{0}^{*}=\frac{x_{0}\left(j\right)-\underset{j}{\min}x_{i}\left(j\right)}{\underset{j}{\max}x_{i}\left(j\right)-\underset{j}{\min}x_{i}\left(j\right)}$$

Determine the *j*-th point’s ${∆}_{0i}\left(j\right)$ distance, or the exact magnitude of the $x_{0}^{*}\left(j\right)-x_{i}^{*}\left(j\right)$ difference. Equation (7) is given as follows:

(7) $${∆}_{0i}\left(j\right)=\left|x_{0}^{*}\left(j\right)-x_{i}^{*}\left(j\right)\right|$$

Using the following Equation (8), apply the Gray relational equation to obtain the Gray relational coefficient $\gamma_{0i}\left(j\right)$:

(8) $$\gamma_{0i}\left(j\right)=\frac{∆min+\xi ∆max}{{∆}_{0i}\left(j\right)+\xi ∆max}$$

where $∆max=\underset{i}{\max}\underset{j}{\max}{∆}_{0i}\left(j\right)$, $∆min=\underset{i}{\min}\underset{j}{\min}{∆}_{0i}\left(j\right),$ and $\xi \in \left[0,1\right]$.

Calculate the *Γ*_0*i*_ value for the Gray coefficient by using Equation (9). The degree of Gray coefficient *Γ*_0*i*_ is calculated as follows if the weights (*W_i_*) of the criterion are established:

(9) $$\Gamma_{0i}=\overset{n}{\underset{j=1}{\sum }}\left[W_{i}\left(j\right)\times r_{0i}\left(j\right)\right]$$

Any alternative that has the highest *Γ*_0*i*_ value is the most significant alternative for decision-making procedures. As a result, the ranking of alternatives’ priority can be determined by the values of *Γ*_0*i*_.

#### 2.3.3. CoCoSo Method

In contrast to these three methods, the CoCoSo method was developed by [59], which combines the concepts of simple additive weighting (SAW), weighted aggregated sum product assessment (WASPAS), and multiplicative exponential weighting (MEW). CoCoSo’s ability to combine data enables the creation of more reliable models and the making of judgments that are more precise [60]. The CoCoSo method is an all-purpose ranking technique that can be applied to a variety of tasks, including supplier selection by [61], waste management by [62], environmental and energy planning by [63], social issues by [64], 5G industries by [65], cost rationalization by [66], location selection by [67], and evaluation of outside service providers by [68], to name a few. The steps of the CoCoSo method are as follows [69]:

After establishing the initial decision matrix, based on the compromise normalization equation, the normalization of criteria values is carried out by using Equation (10) for the benefit-oriented criterion and Equation (11) for the cost-oriented criterion:

(10) $$r_{ij}=\frac{x_{ij}-\underset{i}{\min}x_{ij}}{\underset{i}{\max}x_{ij}-\underset{i}{\min}x_{ij}}$$

(11) $$r_{ij}=\frac{\underset{i}{\max}x_{ij}-x_{ij}}{\underset{i}{\max}x_{ij}-\underset{i}{\min}x_{ij}}$$

Based on the following Equations (12) and (13), determine the sum of weighted comparability (*S_i_*) and power weighted comparability sequences (*P_i_*) for each alternative:

(12) $$S_{i}=\overset{n}{\underset{j=1}{\sum }}\left(w_{j}r_{ij}\right)$$

(13) $$P_{i}=\overset{n}{\underset{j=1}{\sum }}{\left(r_{ij}\right)}^{w_{j}}$$

Develop three aggregated assessment ratings to determine the alternatives’ respective weights by using Equations (14)–(16):

(14) $$k_{ia}=\frac{P_{i}+S_{i}}{\sum_{i=1}^{m}\left(P_{i}+S_{i}\right)}$$

(15) $$k_{ib}=\frac{S_{i}}{\underset{i}{\min}S_{i}}+\frac{P_{i}}{\underset{i}{\min}P_{i}}$$

(16) $$k_{ic}=\frac{\lambda (S_{i})+\left(1-\lambda \right)\left(P_{i}\right)}{\left(\lambda \underset{i}{\max}S_{i}+\left(1-\lambda \right)\underset{i}{\max}P_{i}\right)}$$

In general, Equation (14) reflects the arithmetic mean of the sums of the WSM (weighted sum method) and WPM (weighted product method) scores, whereas Equation (15) denotes the sum of the relative WSM and WPM scores in relation to the best choice. The balanced compromise score of the WSM and WPM models is calculated by Equation (16). Although the value of Equation (16) might range from 0 to 1, 0.50 is typically used as the threshold value.

(17) $$k_{i}={\left(k_{ia}k_{ib}k_{ic}\right)}^{\frac{1}{3}}+\frac{1}{3}\left(k_{ia}+k_{ib}+k_{ic}\right)$$

Using the decreasing order of the total score, determine the final rating of the options (*k_i_*) calculated by using Equation (17).

#### 2.3.4. VIKOR Method

The VIKOR method was created to optimize complex systems based on a number of factors. The compromise solution, compromise ranking list, and weight stability intervals for preference stability of the compromise solution produced with the initial (provided) weights are all determined. This approach concentrates on rating and selecting from a set of options when there are conflicting criteria. It proposes a multi-criterion ranking system based on a specific measure of “closeness” to the “ideal” response [70].

The compromise ranking could be carried out by comparing the measure of closeness to the ideal choice, given that each alternative is evaluated in accordance with each criterion function. The Lp-metric, which is utilized as an aggregating function in a compromise programming approach, forms the basis of the multicriteria measure for compromise ranking [70,71,72].

The following are the steps in the VIKOR compromise ranking algorithm [70]:

Find the best $f_{i}^{*}$ and worst $f_{i}^{-}$ values for each criterion function, where *i* = 1 through *n*. If the ith function provides a benefit, then Equations (18) and (19) are used:

(18) $$f_{i}^{*}=\underset{j}{\max}f_{ij}$$

(19) $$f_{i}^{-}=\underset{j}{\min}f_{ij}$$

Using the relationships, Equations (20) and (21) calculate the values *S_j_* and *R_j_ j* = 1; 2; …; *J*:

(20) $$S_{j}=\overset{n}{\underset{i=1}{\sum }}w_{i}\left(f_{i}^{*}-f_{ij}\right)/\left(f_{i}^{*}-f_{i}^{-}\right)$$

(21) $$R_{j}=\underset{i}{\max}\left[w_{i}\left(f_{i}^{*}-f_{ij}\right)/\left(f_{i}^{*}-f_{i}^{-}\right)\right]$$

where *w_i_* are the criteria weights, indicating their relative weights.

Determine the values of *Q_j_*, *j* = 1, 2, …, *J* using the relation (see Equation (22)):

(22) $$Q_{j}=v\frac{S_{j}-\underset{j}{\min}S_{j}}{\underset{j}{\max}S_{j}-\underset{j}{\min}S_{j}}+\left(1-v\right)\frac{R_{j}-\underset{j}{\min}R_{j}}{\underset{j}{\max}R_{j}-\underset{j}{\min}R_{j}}$$

where *v* represents the weight of the “majority of criteria” strategy (or “the maximum group utility”); in this case, *v* = 0.5.

Alternatives are ranked from the largest to the smallest for each of the three parameters (i.e., *S*, *R*, and *Q*).

If the following two conditions are met, the alternative ($a^{\prime }$), determined as the best, according to the parameter *Q* (minimum), is accepted as a compromise solution:

C1. “Acceptable advantage”:

(23) $$Q\left(a^{\prime \prime }\right)-Q\left(a^{\prime }\right)\geq DQ$$

where ($a^{\prime \prime }$) is the alternative that is ranked second in the list of alternatives by *Q*; *DQ* = 1/(*J* − 1); and *J* means the number of alternatives in Equation (23).

C2. “Acceptable stability in decision-making”:

The best ranking for alternative $a^{\prime }$ must also come from *S* and/or *R*. Within a decision-making process, such as “vote by majority rule” (when *v* > 0.5), “by consensus” (when *v* ≈ 0.5), or “with veto” (when *v* < 0.5), this compromise solution is stable. Thus, *v* denotes the importance of the “majority of criteria” (also known as “the largest group utility”) in the decision-making process [70].

A group of compromise alternatives are suggested in the event that one of the prerequisites is not met. They are as follows:

If only criterion C2 is unsatisfied, then the alternatives are $a^{\prime }$ and $a^{\prime \prime }$; or

If condition C1 is not met, there exist options $a^{\prime }$, $a^{\prime \prime }$, ……, and $a^{m}$, and the value of $a^{m}$, is defined by the relation $Q(a^{\left(M\right)})-Q\left(a^{\prime }\right)<DQ$ for a maximum *M*.

The option with the lowest value of Q is considered to be the best alternative. The compromise ranking list of options and the compromise solution with the “advantage rate” make up the primary ranking outcome [70].

## 3. Results and Discussion

### Criterion Weights

The hierarchical structure for the decision problem handled in this study is shown in Figure 2. Accordingly, 12 alternative polymeric materials are evaluated under 14 different criteria. The selection of the most suitable material for flexible PHP manufacturing was conducted by using three different MCDM methods (i.e., GRA, CoCoSo, and VIKOR). The criteria weights were first calculated by the AHP method, and, thus, the pairwise comparison matrix, which developed as a result of meetings held with the experts working in the related area, is shown in Table 4. The normalized decision matrix and, finally, determined criterion weights are shown in Table 5.

A consistency analysis was performed to make the pairwise comparison matrix acceptable. Table 6 shows the results of the consistency analysis. Accordingly, the consistency index (CI) was calculated at 0.025. The consistency ratio was calculated to be 0.02 by dividing the consistency index by the random consistency index, and it was found to be less than the threshold value of 0.10. Thus, it is understood that the developed pairwise comparison matrix is consistent, and the criterion weights calculated by the AHP method can be easily used in the methods in which the alternatives are listed.

**Figure 3:**
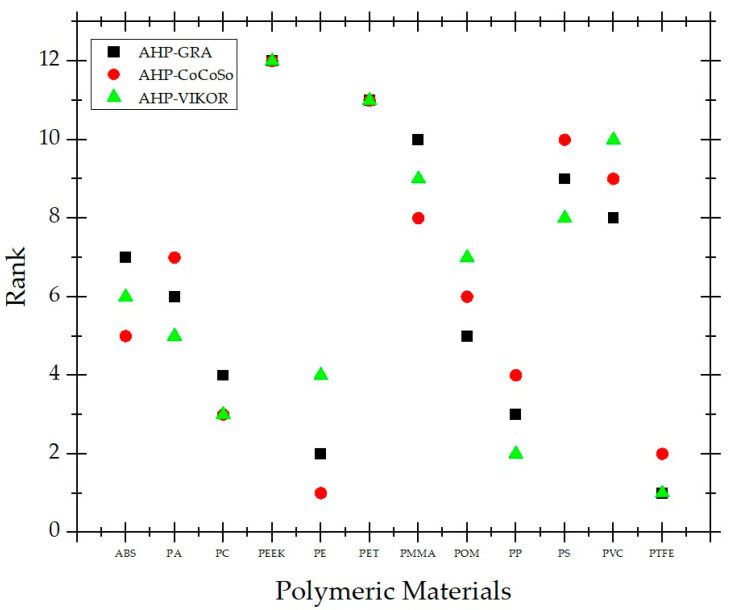
Comparison of rankings from AHP-GRA, AHP-CoCoSo, and AHP-VIKOR approaches.

Three different hybrid MCDM approaches, AHP-GRA, AHP-CoCoSo, and AHP-VIKOR, were successfully used in the current experiment to prioritize the alternatives. The rankings of the alternatives obtained through AHP-GRA, AHP-CoCoSo, and AHP-VIKOR are summarized in Table 7, and the results are plotted in Figure 3. As can be seen from Figure 3 and Table 7, it can be summarized through the three methods that the ranking of material alternatives is basically consistent. As a result, the suggested method is a reasonable and efficient way to assess the performance of alternative material options and choose the best polymeric material.

According to the results of these three methods, PTFE is the most suitable material alternative for the production of flexible pulsating heat pipes. In the AHP-GRA and AHP-VIKOR methods, PTFE is in first place, and, in the AHP-CoCoSo method, it is in second place. Despite its high price compared to many other thermoplastic material alternatives, the main reasons why PTFE is the most suitable material alternatives are its high elongation rate and low Young’s modulus. A further factor that has elevated it to the top is its ability to operate in the extremely low and extremely high temperature ranges anticipated by many heat transfer devices. It is important to confirm our ranking results and to note that some existing studies in the literature show that using PTFE in flexible PHP production will be the correct option [73].

In the AHP-CoCoSo method, PE took first place. It has the lowest Young’s modulus value among alternative materials, after PTFE. This is enough to emphasize that the low Young modulus value is the most important feature expected from a material in flexible PHP production. Unlike AHP-CoCoSo, PE ranked second in AHP-GRA and fourth in AHP-VIKOR. Other important factors in PE’s being in the top ranks are that it has high thermal expansion, high thermal conductivity, and high specific heat values. Thanks to these critical physical, mechanical, and thermal properties advantages, PE has found a place in the production of flexible PHP [12]. Another notable result is that PEEK came in last in all three methods applied. The two most important methods underlying this are the high cost compared to other options and the very high Young’s modulus value. In addition, the material with the highest flexural modulus, which is expected to be as low as possible, is PEEK.

A further noteworthy outcome is PET, another material that is arranged in the same sequence using three separate techniques as PEEK. It was ranked eleventh in all three methods. It possesses practically the lowest thermal conductivity and the lowest possible maximum temperature, which are the main causes of this. Its high flexural modulus and Young’s modulus also caused it to fall behind in the rankings. Polypropylene, which was previously selected for the production of flexible PHPs and serpentine channels, ranked second in the AHP-VIKOR method [38,74]. Close to its position here, it placed third in AHP-GRA and fourth in AHP-CoCoSo. The reasons behind its top ranking are its affordability, high elongation percentage, and low flexural and Young’s modulus values. In addition, the fact that it is a plastic material that can operate at the highest temperature, after PTFE, is an important indicator of how suitable it is for the production of heat transfer devices.

Spearman’s rank correlation analysis was performed to understand how closely MCDM methods produced results with each other in the literature [22,24,75,76,77]. The correlation coefficients between the approaches were thus calculated using Spearman’s rank correlation coefficients. Table 8 displays the results of the Spearman’s rank correlation coefficient calculation for the rankings. These results indicate that there are significant correlations among all of the MCDM strategies taken into consideration. The correlation coefficient between the two MCDM approaches is shown to be larger than 0.9. Moreover, comparing techniques results in higher values of correlation coefficients; i.e., 0.9371 for AHP-GRA versus AHP-VIKIOR, 0.9441 for AHP-GRA versus AHP-VIKOR, and 0.9091 for AHP-CoCoSo versus AHP-VIKOR. The acceptable Spearman’s rank correlation coefficients for the approaches can be deduced at this point.

## 4. Conclusions

Using multi-criteria decision-making techniques, the issue of selecting appropriate polymeric materials for creating flexible PHPs and flexible heat and fluidic devices has been resolved in accordance with their mechanical, physical, and thermal properties. Three multi-criteria decision-making (MCDM) methodologies were used to select twelve thermoplastic materials in a reliable manner for this goal. AHP-GRA, AHP-CoCoSo, and AHP-VIKOR were given an ordered list of possibilities, and the outcomes were compared. The results found similarity in the rankings with the applied hybrid MCDM techniques. The three ideal options are also in this order: Polytetrafluoroethylene (PTFE) > Polyethylene (PE) > Polypropylene (PP). As a result, PTFE has been the best thermoplastic material to be selected among the determined criteria and alternatives. Low flexural and Young’s modulus were the driving factors in this decision. In addition to its high elongation percentage, its ability to work in very wide temperature ranges is one of the most important features that distinguish it from other alternatives.

This research introduced an integrated hybrid multi-criteria decision-making approach to tackle the material selection problem. The literature-based studies that were consulted to support the material selection order were able to demonstrate that it would be practical to use them in the actual manufacturing process. The proposed strategy’s logic and dependability are demonstrated by comparison with the current approach. The efficiency and simplicity of the approach make it applicable to a wider range of material selections and mean it will benefit new researchers in the manufacture of flexible PHPs, as well as other flexible heat and fluidic devices. The study will provide very important benefits to the employees in the manufacturing and academic sectors by reducing the time and labor losses experienced in the material selection process. The study assists designers and industrialists during the selection process of thermoplastic materials to be selected in the production of flexible heat and fluidic devices. Researchers and scientists working in the domains of materials, heat transfer, and fluid mechanics can gain knowledge from the study’s findings and analyses.

The shortcoming of the current study is that a greater variety of thermoplastic materials were not taken into account. The number of thermoplastics used for the present study can be increased in the future. The study can be expanded by considering different thermoplastic materials and polymeric composites. The scope of the study can be further expanded by adding additional properties to the selection parameters in addition to the existing physical, chemical, and thermal properties. Moreover, in the present study, the uncertainty that exists in deciding on material selection, which can be considered a future scope of further research, has been excluded. Thanks to this proposed model, it can also be applied in terms of decision-making in other elements of the design and development of a product. Moreover, further research is needed to use the methodologies in real-world manufacturing settings.

In our study, we used polyethylene (PE) as a general term, covering both high-density polyethylene (HDPE) and low-density polyethylene (LDPE). The properties of HDPE and LDPE are quite different, due to their different molecular structures. HDPE has a linear structure, which makes it sturdy and dense, whereas LDPE has a branched structure, giving it less density and more flexibility. However, in our MCDM analysis, we initially simplified the model to only include major classes of polymeric materials, due to the wide variety of materials available, to reduce complexity. Hence, “PE” was used as a representative term for both types of polyethylene. For a more detailed selection, HDPE and LDPE could be evaluated separately in a future study, as the differences in their properties could have a significant impact on the performance of the flexible pulsating heat pipes.

Within the scope of this research, our primary focus was on the technical aspects of material selection for the manufacturing of flexible pulsating heat pipes. Nonetheless, we absolutely acknowledge the growing importance of environmental considerations in the design and development process of products. In light of this, future iterations of our model could potentially include criteria that account for environmental sustainability, such as recyclability, energy consumption during production, and the overall life-cycle environmental impact. The selection of materials should not only meet the technical requirements, but should also align with sustainability goals. Moreover, for future studies, we could explore biodegradable thermoplastics or thermoplastics derived from renewable sources. This will not only aid in decision-making for product development, but will also direct research and industrial efforts towards more sustainable and environmentally friendly material choices.

## Figures and Tables

**Table 1:** Alternative thermoplastics and abbreviations.

Thermoplastics	Abbreviation
Acrylonitrile butadiene styrene	ABS
Polyamides (Nylons)	PA
Polycarbonate	PC
Polyetheretherketone	PEEK
Polyethylene	PE
Polyethylene terephthalate	PET
Polymethyl methacrylate	PMMA
Polyoxymethylene (Acetal)	POM
Polypropylene	PP
Polystyrene	PS
Polyvinylchloride	PVC
Polytetrafluoroethylene (Teflon)	PTFE

**Table 2:** Average physical, thermal, and mechanical properties for each thermoplastic [47].

Thermoplastics	D	P	E	FM	YM	YS	TS	FT	TM	SH	TC	TE	MINT	MAXT
ABS	1100	2.55	50.00	2.50	2.00	35.00	42.00	2.80	110.00	1650	0.27	160.00	−20	80
PA	1100	4.30	65.00	2.30	2.90	72.00	130.00	3.90	50.00	1650	0.24	145.00	−30	95
PC	1150	4.85	100.0	2.30	2.20	65.00	66.00	3.40	170.00	1550	0.21	130.00	−30	120
PEEK	1300	97.0	90.00	3.80	3.90	80.00	85.00	3.50	170.00	1450	0.25	130.00	−65	70
PE	950	2.20	15.00	0.80	0.76	23.50	33.00	1.60	125.00	1850	0.42	165.00	−40	130
PET	1350	2.20	20.00	3.30	3.45	59.50	60.00	5.00	75.00	1450	0.15	115.00	−30	60
PMMA	1200	2.85	2.50	2.90	3.00	63.00	64.00	1.20	125.00	1550	0.17	115.00	−70	70
POM	1400	3.15	40.00	2.83	3.75	61.00	75.00	2.90	170.00	1400	0.29	140.00	−30	80
PP	900	2.25	120.0	1.50	1.25	29.00	35.00	3.80	160.00	1950	0.14	150.00	−20	160
PS	1050	3.15	30.00	2.50	1.90	43.00	47.00	0.90	90.00	1750	0.13	120.00	−50	70
PVC	1450	2.10	1.60	3.00	3.10	43.00	53.00	3.30	88.00	1400	0.22	125.00	−20	60
PTFE	2150	16.0	300.0	0.60	0.48	20.00	25.00	1.60	115.00	1100	0.25	175.00	−100	260

**Table 3:** Criteria and abbreviations.

Thermoplastics	Units	Abbreviation
Density	kg/m^3^	D
Price	US $/kg	P
Elongation	%	E
Flexural modulus	GPa	FM
Young’s modulus	GPa	YM
Yield strength	MPa	YS
Tensile strength	MPa	TS
Fracture toughness	MPa.m^1/2^	FT
Melting temperature or glass transition	°C	TM
Specific heat capacity	J/kg. °C	SH
Thermal conductivity	W/m/K	TC
Thermal expansion	10^−6^/°C	TE
Minimum temperature	°C	MINT
Maximum temperature	°C	MAXT

**Table 4:** Pairwise comparison matrix of the criteria.

	D	P	E	FM	YM	YS	TS	FT	TM	SH	TC	TE	MINT	MAXT
D	1	1/9	1/6	1/4	1/9	1/6	1/6	1/3	1/7	1/6	1/8	1/6	1/7	1/9
P	9	1	3	5	1	3	3	6	2	3	2	3	2	1
E	6	1/3	1	2	1/3	1	1	3	2	1	1/2	1	2	1/3
FM	4	1/5	1/2	1	1/5	1/2	1/2	2	1/3	1/2	1/4	1/2	1/3	1/5
YM	9	1	3	5	1	3	3	6	2	3	2	3	2	1
YS	6	1/3	1	2	1/3	1	1	3	1/2	1	1/2	1	1/2	1/3
TS	6	1/3	1	2	1/3	1	1	3	1/2	1	1/2	1	1/2	1/3
FT	3	1/6	1/3	1/2	1/6	1/3	1/3	1	1/4	1/3	1/5	1/3	1/4	1/6
TM	7	1/2	1/2	3	1/2	2	2	4	1	2	1/2	2	1	1/2
SH	6	1/3	1	2	1/3	1	1	3	1/2	1	1/2	1	1/2	1/3
TC	8	1/2	2	4	1/2	2	2	5	2	2	1	2	2	1/2
TE	6	1/3	1	2	1/3	1	1	3	1/2	1	1/2	1	1/2	1/3
MINT	7	1/2	1/2	3	1/2	2	2	4	1	2	1/2	2	1	1/2
MAXT	9	1	3	5	1	3	3	6	2	3	2	3	2	1

**Table 5:** Normalized matrix and criteria weights.

	D	P	E	FM	YM	YS	TS	FT	TM	SH	TC	TE	MINT	MAXT	Weights (%)
D	0.01	0.02	0.01	0.01	0.02	0.01	0.01	0.01	0.01	0.01	0.01	0.01	0.01	0.02	1.05
P	0.10	0.15	0.17	0.14	0.15	0.14	0.14	0.12	0.14	0.14	0.18	0.14	0.14	0.15	14.35
E	0.07	0.05	0.06	0.05	0.05	0.05	0.05	0.06	0.14	0.05	0.05	0.05	0.14	0.05	6.43
FM	0.05	0.03	0.03	0.03	0.03	0.02	0.02	0.04	0.02	0.02	0.02	0.02	0.02	0.03	2.83
YM	0.10	0.15	0.17	0.14	0.15	0.14	0.14	0.12	0.14	0.14	0.18	0.14	0.14	0.15	14.35
YS	0.07	0.05	0.06	0.05	0.05	0.05	0.05	0.06	0.03	0.05	0.05	0.05	0.03	0.05	4.98
TS	0.07	0.05	0.06	0.05	0.05	0.05	0.05	0.06	0.03	0.05	0.05	0.05	0.03	0.05	4.98
FT	0.03	0.03	0.02	0.01	0.03	0.02	0.02	0.02	0.02	0.02	0.02	0.02	0.02	0.03	1.99
TM	0.08	0.08	0.03	0.08	0.08	0.10	0.10	0.08	0.07	0.10	0.05	0.10	0.07	0.08	7.59
SH	0.07	0.05	0.06	0.05	0.05	0.05	0.05	0.06	0.03	0.05	0.05	0.05	0.03	0.05	4.98
TC	0.09	0.08	0.11	0.11	0.08	0.10	0.10	0.10	0.14	0.10	0.09	0.10	0.14	0.08	9.72
TE	0.07	0.05	0.06	0.05	0.05	0.05	0.05	0.06	0.03	0.05	0.05	0.05	0.03	0.05	4.98
MINT	0.08	0.08	0.03	0.08	0.08	0.10	0.10	0.08	0.07	0.10	0.05	0.10	0.07	0.08	7.42
MAXT	0.10	0.15	0.17	0.14	0.15	0.14	0.14	0.12	0.14	0.14	0.18	0.14	0.14	0.15	14.35

**Table 6:** Consistency ratios.

Parameters	Values
Number of comparisons	14
Average consistency (*λ_max_*)	14.33
Consistency index (*CI*)	0.025
Random consistency index (*RI*)	1.57
Consistency ratio (*CR*)	0.02

**Table 7:** Rankings of alternative polymeric materials for flexible PHPs manufacturing by hybrid MCDM approaches.

Thermoplastics	AHP-GRA		AHP-CoCoSo					AHP-VIKOR			
	*Γ*_0*i*_	Rank	*k_a_*	*k_b_*	*k_c_*	*k*	Rank	*S_i_*	*R_i_*	*Q*_0_	Rank
ABS	0.034	7	0.091	2.879	0.968	1.946	5	0.547	0.129	0.704	6
PA	0.035	6	0.086	2.795	1.017	1.924	7	0.548	0.118	0.643	5
PC	0.037	4	0.093	3.112	1.021	2.075	3	0.486	0.101	0.457	3
PEEK	0.030	12	0.072	2.053	1.004	1.572	12	0.707	0.144	1.000	12
PE	0.044	2	0.092	3.321	1.026	2.160	1	0.419	0.136	0.576	4
PET	0.031	11	0.076	2.134	1.005	1.618	11	0.703	0.144	0.995	11
PMMA	0.032	10	0.087	2.530	1.010	1.816	8	0.635	0.136	0.862	9
POM	0.035	5	0.090	2.775	1.015	1.926	6	0.573	0.137	0.785	7
PP	0.041	3	0.087	3.097	1.023	2.053	4	0.461	0.094	0.386	2
PS	0.032	9	0.077	2.392	1.010	1.730	10	0.630	0.136	0.855	8
PVC	0.033	8	0.079	2.383	1.010	1.731	9	0.639	0.144	0.910	10
PTFE	0.049	1	0.071	3.285	1.033	2.084	2	0.331	0.057	0.0000	1

**Table 8:** The Spearman’s rank correlation coefficients for the hybrid MCDM approaches used in the study.

	AHP-GRA	AHP-CoCoSo	AHP-VIKOR
AHP-GRA	1.0000	0.9441	0.9371
AHP-CoCoSo		1.0000	0.9091
AHP-VIKOR			1.0000

## Data Availability

Data sharing not applicable.
